# Clinical Management of Hemodialyzed Patients: From Pharmacological Interventions to Advanced Technologies

**DOI:** 10.3390/jcm11154310

**Published:** 2022-07-25

**Authors:** Paolo Monardo, Antonio Lacquaniti

**Affiliations:** Nephrology and Dialysis Unit, Department of Internal Medicine, Papardo Hospital, 98158 Messina, Italy; ant.lacq@gmail.com

Morbidity and mortality have marginally decreased over the last 3 decades in hemodialyzed (HD) patients, despite multiple pharmacological and technological interventions. Randomized trials failed to demonstrate the superiority of one procedure, schedule, or pharmacological intervention on survival and, consequently, best practice guidelines revealed a weak level of recommendations [1].

The complexity of uremia, characterized by several metabolic disorders and risk factors influencing all-cause mortality, explains these negative results. The consequence is that specific and direct interventions fail to improve or impact patient outcomes. Chronic systemic inflammation contributes to atherosclerosis, cardiovascular (CV) disease, and anemia. Furthermore, immune deficiency leads to impaired response to vaccination and increased incidence, severity, and poor outcome of microbial infections [2].

Moreover, in HD patients, the accumulation of uremic toxins, molecules exerting adverse biological activity at high concentrations, affects the patients’ survival rate and quality of life. Up to date, personalization represents a better option for dialysis recommendation, according to the heterogeneity of HD patients, based on their dominant comorbidities, clinical characteristics, and parameters related to HD prescription.

The great concern in the clinical management of HD patients is related to the control and correction of weight gain, dyslipidemia, hypertension, and anemia, the latter caused by endogenous erythropoietin (ESA) deficiency and resistance and by low-serum iron levels [3]. Moreover, secondary hyperparathyroidism (sHPT) is another frequently observed complication in these patients, characterized by mineral bone disorders associated with vascular calcifications and consequent CV disease [4]. 

Fluid volume and hemodynamic management are essential components of dialysis adequacy. Whereas restoring salt and water homeostasis, reaching the right dry weight, has been associated with benefits on CV outcome, on the other hand, the stressful fluid removal during intermittent HD was associated with CV disease and organ damage. 

This high-risk population requires a more precise approach. 

Behind the clinical assessment, non-invasive instrumental tools could provide direct handling of sodium and water in a more precise and personalized way. 

Lung ultrasound provides reliable estimates of extravascular water in the lung, a critical parameter of the central circulation reflecting the left ventricular end-diastolic pressure [5].

Advances in microelectronics have enabled uni- and multiparameter wearable sensors capable of assessing fluid volume, with significant progress in predicting complex volume-related events during dialysis, such as blood hypotension, using machine learning [6].

Circulating cardiac biomarkers, such as natriuretic peptides, represent additional methods to evaluate hydration status in HD patients. Observational studies suggest a significant association of these peptides with volume status, assessed with bio-impedance analysis, and mortality [7,8].

Moreover, the real-time evaluation of the blood volume, through biofeedback systems connected to the dialysis machine, could assess the volume status, monitoring and guiding fluid removal during each dialysis session [9].

Moderate or severe intradialytic hypotension is related to myocardial ischemia and stress-induced myocardial dysfunction, as well as there exists a relationship between pre- to post-dialysis changes in blood pressure and mortality. At the same time, volume overload is associated with blood hypertension and cardiac dysfunction, representing a risk factor for all-cause and CV mortality in this population.

In the near future, these tools will support nephrologist decision-making with a high potential for improving CV outcomes.

Anemia management represents another key aim to be achieved in HD patients, obtaining high hemoglobin, transferrin saturation, and ferritin levels in combination with significantly low doses of ESA and low erythropoietin-resistance index [10].

Anemia is associated with morbidity and mortality in chronic kidney disease. The use of ESA is related to an improved functional status, quality of life, and lower requirements for blood transfusion, but it constitutes a large share of the costs of injectable drugs. Moreover, high doses of ESA in patients with comorbidities have been associated with worse outcomes in randomized controlled trials [11].

Hypoxia-inducible factor (HIF) prolyl hydroxylase inhibitors (HIF-PHIs) are new drugs that mimic the response to moderate hypoxia, increasing the production of endogenous erythropoietin in the kidneys and, to a lesser extent, in the liver. Roxadustat, the first-in-class HIF-PH inhibitor, stabilizes the HIF-alfa subunit and promotes an erythropoietic response, including increased synthesis of endogenous erythropoietin, iron absorption, transport, and mobilization [12]. Roxadustat effectively increased hemoglobin in HD patients requiring minimum iron supplementation, revealing efficacy in patients with inflammation, a common cause of ESA hyporesponsiveness [13].

However, despite these encouraging findings, HIF-PHIs are still surrounded by some uncertainties, partly because the HIF pathway is complex and not fully explored and understood, with potential pleiotropic effects involving other pathways, such as the Vascular Endothelium Growth Factor, with potentially negative consequences, such as endothelial dysfunction, vascular thrombosis, and cancer development [14].

The clinical management of these patients is more difficult if the mineral bone disorder occurs. sHPT results in high-turnover bone, bone mineral loss, calcification of soft tissues including arteries, and low parathyroid hormone (PTH) levels may indicate adynamic bone disease, which weakens the buffering capacity of bone for fluctuations in serum phosphorus and calcium, increasing vascular calcification and mortality rates. HD patients with low PTH and those with sHPT have similar long-term outcomes in terms of all-cause death and cardio-cerebrovascular death [15]. In this context, vitamin D receptor activators and calcimimetics (calcium-sensing receptor agonists) represent the two major options for medical treatment. Recently, several studies demonstrated the efficacy of etelcalcetide in the control of sHPT, with higher treatment adherence and persistence if compared to cinacalcet, with disease-specific cost savings related to drugs and hospitalizations [16].

However, behind PTH and phosphate levels, other bone-related biomarkers, such as fibroblast growth factor-23 and fetuin-A, play important roles in vascular calcification, revolutionizing the understanding of Calcium–Phosphate balance regulation and changing the once-simplistic view of the more complex, multi-organ feedback system, acting to maintain the physiological concentrations of calcium and phosphate [17].

Moreover, all these bone-derived products, whose levels achieve even 1000-fold above the physiological limit in HD patients, belong to the group of uremic toxins. Current strategies decrease their serum concentration through dietary and pharmacological interventions, mainly via modulation of intestinal absorption capacity through binding effects and/or reduction of ingested amounts of the toxins or their precursors, and by extra-corporeal removal [18,19].

Convective dialytic strategies improve the removal of middle molecules and protein-bound uremic toxins. Hemodiafiltration (HDF) enhanced uremic toxin removal, such as beta2-microglobulin, with consequent clinical benefits, whereas contrasting data are reported about phosphate removal [20].

However, the beneficial effects of HDF on inflammation and anemia have been revealed, with a significant improvement of several biomarkers linked to the better removal of middle-sized uremic toxins and inflammatory mediators [21,22]. To achieve positive effects using this technique, a blood flow over 300 mL/min is required for obtaining sufficient rates of ultrafiltration, acceptable transmembrane pressure gradients, and high volumes of reinfusion. The reduced mortality risk has been inversely correlated with the magnitude of the convection volume, with positive effects observed if post-dilutional convective volume doses were >23 L/session [23].

Recent advances in the membrane manufacturing industry have led to the development of a novel class of medium cut-off (MCO) dialyzers applied to standard bicarbonate dialysis, which could better reduce the circulating levels of middle molecules while allowing albumin to remain in the plasma, demonstrating non-inferiority results when compared with HDF [24].

However, we need further studies with major CV events or death as a primary outcome, comparing different dialytic strategies.

In the field of membrane engineering, double-layer mixed-matrix membranes, which combine dialysis and adsorption, have shown improved biocompatibility and a better removal of protein-bound uraemic toxins. The use of dialyzer membranes coated with bioactive compounds has also been proposed to further ameliorate inflammation and oxidative stress. Moreover, the inclusion of renal epithelial cells may open a new scenario with an active role of the membrane, waiting for kidney organoid technologies.

## References

[1] Monardo P., Lacquaniti A., Campo S., Bucca M., Casuscelli di Tocco T., Rovito S., Ragusa A., Santoro A. (2021). Updates on hemodialysis techniques with a common denominator: The personalization of the dialytic therapy. Semin. Dial..

[2] Campo S., Lacquaniti A., Trombetta D., Smeriglio A., Monardo P. (2022). Immune System Dysfunction and Inflammation in Hemodialysis Patients: Two Sides of the Same Coin. J. Clin. Med..

[3] Visconti L., Benvenga S., Lacquaniti A., Cernaro V., Bruzzese A., Conti G., Buemi M., Santoro D. (2016). Lipid disorders in patients with renal failure: Role in cardiovascular events and progression of chronic kidney disease. J. Clin. Transl. Endocrinol..

[4] Savica V., Santoro D., Monardo P., Mallamace A., Bellinghieri G. (2008). Sevelamer carbonate in the treatment of hyperphosphatemia in patients with chronic kidney disease on hemodialysis. Ther. Clin. Risk Manag..

[5] Zoccali C., Mallamaci F., Picano E. (2022). Detecting and Treating Lung Congestion with Kidney Failure. Clin. J. Am. Soc. Nephrol. CJASN.

[6] Barbieri C., Cattinelli I., Neri L., Mari F., Ramos R., Brancaccio D., Canaud B., Stuard S. (2019). Development of an artificial intelligence model to guide the management of blood pressure, fluid volume, and dialysis dose in end-stage kidney disease patients: Proof of concept and first clinical assessment. Kidney Dis..

[7] Bolignano D., Coppolino G., Romeo A., Lacquaniti A., Buemi M. (2010). Neutrophil gelatinase-associated lipocalin levels in chronic haemodialysis patients. Nephrology.

[8] Harrison T.G., Shukalek C.B., Hemmelgarn B.R., Zarnke K.B., Ronksley P.E., Iragorri N., Graham M.M., James M.T. (2020). Association of NT-proBNP and BNP With Future Clinical Outcomes in Patients With ESKD: A Systematic Review and Meta-analysis. Am. J. Kidney Dis..

[9] Loutradis C., Sarafidis P.A., Ferro C.J., Zoccali C. (2021). Volume overload in hemodialysis: Diagnosis, cardiovascular consequences, and management. Nephrol. Dial. Transpl..

[10] Lacquaniti A., Pasqualetti P., Casuscelli di Tocco T., Campo S., Rovito S., Bucca M., Ragusa A., Monardo P. (2020). Ferric carboxymaltose versus ferric gluconate in hemodialysis patients: Reduction of erythropoietin dose in 4 years of follow-up. Kidney Res. Clin. Pract..

[11] Singh A.K., Szczech L., Tang K.L., Barnhart H., Sapp S., Wolfson M., Reddan D. (2006). Correction of anemia with epoetin alfa in chronic kidney disease. N. Engl. J. Med..

[12] Abdelazeem B., Abbas K.S., Shehata J., El-Shahat N.A., Baral N., Savarapu P., Kunadi A. (2021). The efficacy of Roxadustat for the treatment of anemia in dialysis dependent chronic kidney disease patients: An updated systematic review and meta-analysis of randomized clinical trials. Ann. Transl. Med..

[13] Fishbane S., Pollock C.A., El-Shahawy M., Escudero E.T., Rastogi A., Van B.P., Frison L., Houser M., Pola M., Little D.J. (2022). Roxadustat Versus Epoetin Alfa for Treating Anemia in Patients with Chronic Kidney Disease on Dialysis: Results from the Randomized Phase 3 ROCKIES Study. J. Am. Soc. Nephrol..

[14] Locatelli F., Del Vecchio L. (2022). The Search for the Perfect Agent for Anemia Management in Chronic Kidney Disease. J. Am. Soc. Nephrol..

[15] Guo W., Zhang H., Zhang Y., Huang H., Liu W., Diao Z. (2022). Low Parathyroid Hormone Versus Secondary Hyperparathyroidism and Survival in Patients Undergoing Hemodialysis: A Propensity-Matched Analysis. Front. Endocrinol..

[16] Perrone V., Dovizio M., Veronesi C., Andretta M., Bartolini F., Cavaliere A., Ferrante F., Lupi A., Pagliaro R., Pagnotta R. (2022). Real-World Evaluation of Calcimimetics for the Treatment of Secondary Hyperparathyroidism in Chronic Kidney Disease, in an Italian Clinical Setting. Healthcare.

[17] D’Arrigo G., Mallamaci F., Pizzini P., Leonardis D., Tripepi G., Zoccali C. (2022). CKD-MBD Biomarkers and CKD Progression: An Analysis by the Joint Model. Nephrol. Dial. Transpl..

[18] Savica V., Calò L.A., Monardo P., Santoro D., Mallamace A., Muraca U., Bellinghieri G. (2009). Salivary phosphorus and phosphate content of beverages: Implications for the treatment of uremic hyperphosphatemia. J. Ren. Nutr..

[19] Ehlerding G., Ries W., Kempkes-Koch M., Ziegler E., Erlenkötter A., Zawada A.M., Kennedy J.P., Ottillinger B., Stauss-Grabo M., Lang T. (2021). Randomized comparison of three high-flux dialyzers during high-volume online hemodiafiltration-the comPERFORM study. Clin. Kidney J..

[20] Lin C.L., Yang C.W., Chiang C.C., Chang C.T., Huang C.C. (2001). Long-term on-line hemodiafiltration reduces predialysis beta-2-microglobulin levels in chronic hemodialysis patients. Blood Purif..

[21] Pedrini L.A., Comelli M., Ruggiero P., Feliciani A., Manfrini V., Cozzi G., Castellano A., Pezzotta M., Gatti G., Arazzi M. (2020). Mixed hemodiafiltration reduces erythropoiesis stimulating agents requirement in dialysis patients: A prospective randomized study. J. Nephrol..

[22] Guedes M., Vernooij R.W.M., Davenport A., Kuhlmann M.K., Aregger F., Pecoits-Filho R. (2022). Clinical performance, intermediate and long-term outcomes of high-volume hemodiafiltration in patients with kidney failure. Semin. Dial..

[23] Peters S., Bots M.L., Canaud B., Davenport A., Grooteman M.P., Kircelli F., Locatelli F., Maduell F., Morena M., Nubé M.J. (2016). Haemodiafiltration and mortality in end-stage kidney disease patients: A pooled individual participant data analysis from four randomized controlled trials. Nephrol. Dial. Transpl..

[24] Dellepiane S., Marengo M., D’Arezzo M., Donati G., Fabbrini P., Lacquaniti A., Ronco C., Cantaluppi V. (2022). The Next Evolution of HemoDialysis eXpanded: From a Delphi Questionnaire-Based Approach to the Real Life of Italian Dialysis Units. Blood Purif..

